# Integrated Cross-Scale Manipulation and Modulable Encapsulation of Cell-Laden Hydrogel for Constructing Tissue-Mimicking Microstructures

**DOI:** 10.34133/research.0414

**Published:** 2024-07-16

**Authors:** Yanfeng Zhao, Xinyi Dong, Yang Li, Juan Cui, Qing Shi, Hen-Wei Huang, Qiang Huang, Huaping Wang

**Affiliations:** ^1^ Intelligent Robotics Institute, School of Mechatronical Engineering, Beijing Institute of Technology, Beijing 100081, China.; ^2^ Peking University First Hospital, Xicheng District, Beijing 100034, China.; ^3^ Key Laboratory of Instrumentation Science and Dynamic Measurement, Ministry of Education, North University of China, Taiyuan 030051, China.; ^4^ Beijing Advanced Innovation Center for Intelligent Robots and Systems, Beijing Institute of Technology, Beijing 100081, China.; ^5^ Laboratory for Translational Engineering, Harvard Medical School, Cambridge, MA 02139, USA.; ^6^ Key Laboratory of Biomimetic Robots and Systems (Beijing Institute of Technology), Ministry of Education, Beijing 100081, China.

## Abstract

Engineered microstructures that mimic in vivo tissues have demonstrated great potential for applications in regenerative medicine, drug screening, and cell behavior exploration. However, current methods for engineering microstructures that mimic the multi-extracellular matrix and multicellular features of natural tissues to realize tissue-mimicking microstructures in vitro remain insufficient. Here, we propose a versatile method for constructing tissue-mimicking heterogeneous microstructures by orderly integration of macroscopic hydrogel exchange, microscopic cell manipulation, and encapsulation modulation. First, various cell-laden hydrogel droplets are manipulated at the millimeter scale using electrowetting on dielectric to achieve efficient hydrogel exchange. Second, the cells are manipulated at the micrometer scale using dielectrophoresis to adjust their density and arrangement within the hydrogel droplets. Third, the photopolymerization of these hydrogel droplets is triggered in designated regions by dynamically modulating the shape and position of the excitation ultraviolet beam. Thus, heterogeneous microstructures with different extracellular matrix geometries and components were constructed, including specific cell densities and patterns. The resulting heterogeneous microstructure supported long-term culture of hepatocytes and fibroblasts with high cell viability (over 90%). Moreover, the density and distribution of the 2 cell types had significant effects on the cell proliferation and urea secretion. We propose that our method can lead to the construction of additional biomimetic heterogeneous microstructures with unprecedented potential for use in future tissue engineering applications.

## Introduction

Natural tissues and organs typically comprise multiple types of cells that are embedded in the corresponding extracellular matrix (ECM) based on the specific cell distribution [1,2]. These differences in structural and physicochemical features result in tissues and organs having unique and complex functions. Inspired by this biological feature, tissue engineering has employed cell-laden hydrogels to construct hydrogel microstructures for tissue replication in vitro [3–9]. However, existing technologies for biomimetic tissue fabrication are incapable of satisfactorily replicating the multi-ECM and multicellular distribution features of natural tissues within heterogeneous microstructures. Several studies have demonstrated that modifying certain features, such as the ECM geometry [10,11], component, cell type, density, and pattern, significantly affects the physiological behavior of tissues and organs. For instance, alterations in ECM mechanical properties can affect cell proliferation, differentiation, and migration [12–14]. Differences in cell type or density can affect intercellular communication [15–17], while the arrangement of cells can affect the mechanical properties and regeneration growth of tissues [18–20]. Therefore, the construction of heterogeneous microstructures that mimic the ECM geometry and component, cell type, and density and pattern of natural tissues is highly important for advancing tissue and organ development in vitro.

To construct heterogeneous microstructures that mimic the ECM geometry, component, cell type, and density and pattern in natural tissues, various biofabrication approaches [21–24] have been developed. Among these approaches, bioprinting has attracted considerable attention for its excellent ability to incorporate cell-laden hydrogels and to use controllable patterns to rapidly construct tissue-mimicking microstructures [25–29] . Conventional bioprinting uses a single cell-laden hydrogel, which enables the construction of high-precision microstructures resembling the ECM geometry of natural tissues but often falls short in replicating the heterogeneity of ECM components in natural tissues. In response to this limitation, researchers have explored alternative methods, including the integration of microfluidic [30–32], coaxial nozzle [33,34], and multinozzle [35,36] auxiliary tools, for bioprinting. During such printing, these approaches enable the exchange of multiple cell-laden hydrogels to construct heterogeneous microstructures that replicate the heterogeneous and anisotropic distributions of ECM geometry, components, cell types, and density in natural tissues. However, the random mixing of cells and hydrogels makes it difficult for these methods to manipulate single cells in hydrogels, resulting in random encapsulation of cells in heterogeneous microstructures. Therefore, constructing heterogeneous microstructures that mimic the cell pattern in natural tissues remains very challenging.

To manipulate cells in a liquid environment, various micromanipulation techniques, including optical tweezers [37–39], optoelectronic tweezers [40,41], acoustic tweezers [42–44], dielectrophoresis (DEP) [45,46], and magnetic tweezers [47–49], have been developed. For example, optical tweezers generate a local light field gradient on the surface or inside the cell by focusing the laser beam. This approach creates an optical trap that can move and adjust in 3-dimensional space, allowing for precise manipulation of the position of a single cell or group of cells. Acoustic tweezers use the acoustic radiation force generated by the interaction of the incident acoustic field with the scattered acoustic field from cells to manipulate cells. In a liquid environment, the balance between the acoustic radiation force and the liquid drag force controls the motion of the cells, allowing them to be relocated at certain positions to form various patterns. Using these micromanipulation techniques, a relatively stable mechanical state is established between the microscopic cell and the macroscopic liquid through the action of physical fields (light, sound, electricity, and magnetism) to maintain the stability of the cell position. However, this state is very fragile in the liquid environment and can be disrupted by disturbances, leading to changes in cell position. Exchanging macroscopic liquids is nearly impossible in this relatively stable state. To achieve a steady state from a relatively stable state, researchers have used ultraviolet (UV) exposure [50] or other methods [51,52] to induce a cross-linking reaction in the liquid, which transforms the material into a solid to stably encapsulate the cells. The unregulated nature of the encapsulation process results in the complete transformation of the liquid environment into a solid environment, resulting in the construction of a componential uniform microstructure with cell patterns. However, natural tissues are composed of complex microstructures with nested ECM geometries, heterogeneous ECM components, and anisotropic cell types, densities, and patterns. Replicating these features in vitro to construct tissue-mimicking microstructures remains a challenge. Therefore, there is an urgent need for a method that integrates an orderly combination of macroscopic hydrogel exchange, microscopic cell manipulation, and modulable encapsulation. This method enables the arrangement of cells within hydrogels, controlled encapsulation of hydrogels, and exchange of hydrogels, which can be manipulated repeatedly to construct tissue-mimicking microstructures that replicate the features of natural tissues.

In this study, we present a versatile method for the orderly cross-scale manipulation of hydrogel droplets and cells, as well as a modulable encapsulation technique for realizing tissue-mimicking heterogeneous microstructures. The innovative system integrates an electric force-driven manipulation setup with digital light processing (DLP) bioprinting. Various hydrogel droplets are efficiently manipulated at the millimeter scale using electrowetting on dielectric (EWOD) within a digital microfluidic chip (DMF). The density and arrangement of cells or microparticles within these hydrogel droplets were adjusted at the micrometer scale using DEP. Subsequently, UV light patterns are dynamically adjusted by a digital micromirror device (DMD) for selective photopolymerization of hydrogel droplets, which encapsulate a specific distribution of cells or microparticles into heterogeneous microstructures. We demonstrated the efficient manipulation of 2 typical poly (ethylene glycol) diacrylate (PEGDA) and gelatin methacrylate (GelMA) hydrogel droplets, enabling adjustable density and arrangement of the cells or microparticles. Various heterogeneous microstructures featuring distinct cell or microparticle densities and arrangements are then constructed by photopolymerization. Furthermore, cell assays showed that cells in heterogeneous microstructures maintained favorable viability. The density and distribution of cells significantly impacted cell proliferation and functional expression. The proposed method, which can replicate the ECM geometry, component, cell type, and density and pattern features of in vivo tissues, shows outstanding potential for regenerating complex tissues with physiological relevance in the tissue engineering field.

## Results

### System setup

**Fig. 1.**
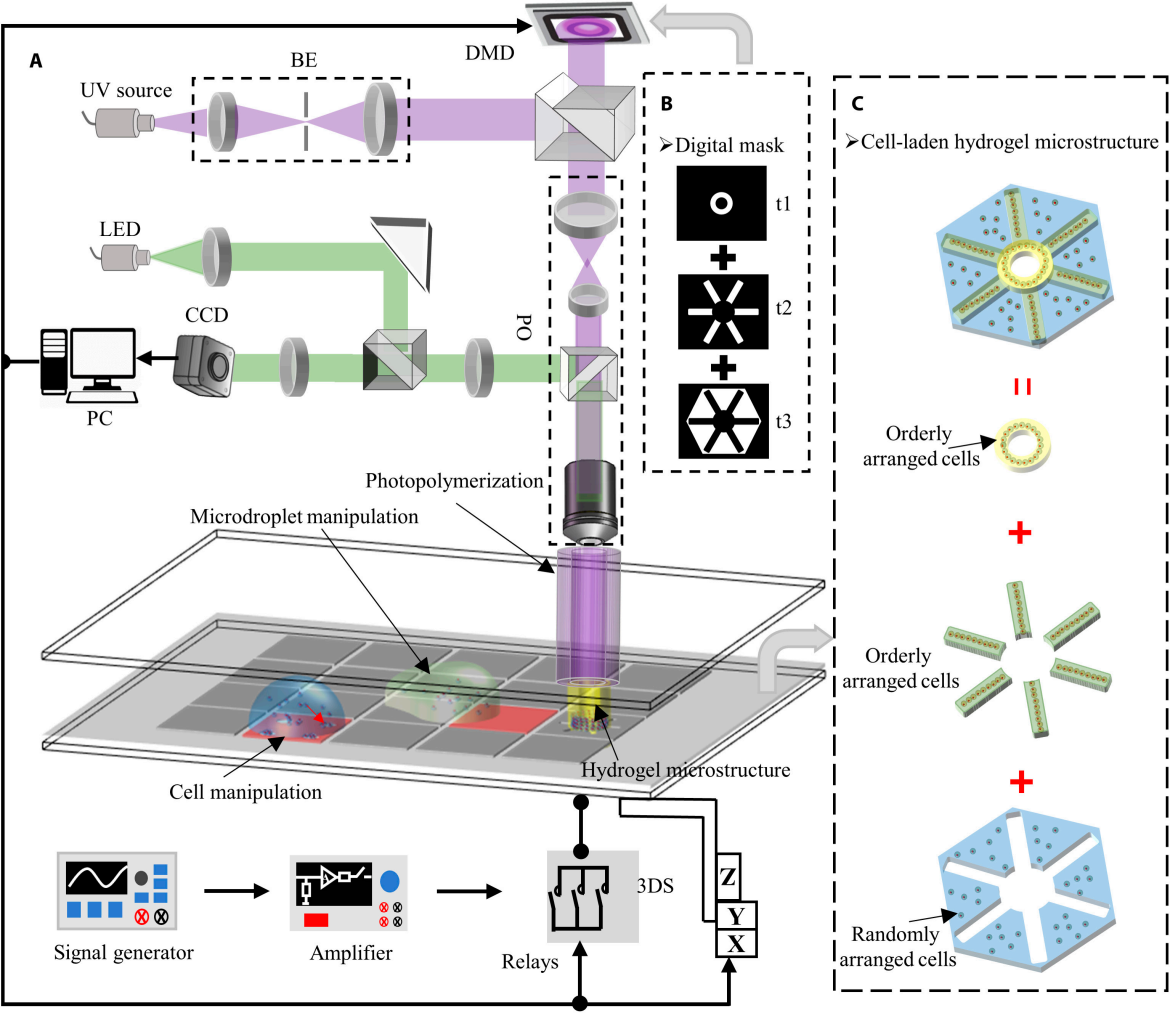
Composite printing system. (A) Schematic diagram of the composite printing system. (B) User-customized digital mask. (C) Biomimetic cell-laden hydrogel microstructure. BE, beam expansion optics; PO, projection optics; CCD, charge-coupled device; PC, personal computer.

The investigated system integrates cross-scale manipulation of hydrogel droplets and cells with modulable encapsulation of hydrogel microstructures. This system comprises 2 major components: electric field force-based manipulation for moving hydrogel droplets and cells and DLP for photopolymerizing hydrogel microstructures using DMD (Fig. 1A). Electric field force-based manipulation was achieved via DMF consisting of 2 plates made of indium tin oxide (ITO)-coated glass slides. The top plate features a continuous ITO coating serving as the ground electrode. The ITO coating on the bottom plate was wet etched to yield individually addressable electrodes, which were covered with a dielectric layer to provide insulation and protection. Both plates were coated with a hydrophobic layer to achieve a high contact angle, ensuring the desired fluid behavior and minimizing surface contamination. The DMF chip was assembled by adhering the top and bottom plates to double-sided tape to form a chamber. The hydrogel droplets and cells were manipulated within this chamber by applying specific electrical signals to individual addressable electrodes on the DMF, generating a localized electric field to induce EWOD or DEP. Hydrogel droplets were driven by EWOD along individually addressable electrodes, allowing for the manipulation of hydrogel droplets in various ways, including their generation, transportation, splitting, and merging on DMF. In contrast, the cells were manipulated by DEP within hydrogel droplets, which enabled adjustment of the cell density or arrangement in the hydrogel droplets. Compared to alternative hydrogel delivery technologies such as continuous microfluidics and hydrogel change cartridges, the electric field force-based manipulation system demonstrated superior flexibility, efficient hydrogel droplet manipulation [53,54], and the ability to manipulate cells within hydrogel droplets.

In the DLP system, a high-power UV beam is appropriately tuned to the desired beam diameter using beam-expanding optics. Then, this beam is propagated onto the surface of the DMD at an angle of 66°. The surface of the DMD comprises hundreds of thousands of aluminum micromirrors (1,024 × 768). When a digital mask (Fig. 1B), which is a binary image designed using PowerPoint software, is input into the DMD, each aluminum micromirror can be dynamically modulated into an ON state and an OFF state according to the corresponding black and white pixels in the digital mask. This modulation technique enables the DMD to selectively reflect the incident light beam, generating the desired light pattern. To ensure the precision of the microstructure, the light pattern is projected onto the hydrogel droplets using projection optics. When exposed to UV light, photopolymerization occurs within the hydrogel droplets, resulting in the formation of microstructures with corresponding shapes. After polymerization, the cells are immobilized and encapsulated in the hydrogel microstructures. The integrated system includes 2 electrical manipulations to drive hydrogel droplets to specific locations and adjust the density or arrangement of cells within the hydrogel droplets. Subsequently, a dynamic light pattern is used to control the photopolymerization of the hydrogel droplets, enabling the construction of programmable hydrogel microstructures with controllable geometries, components, cell densities/types, and cell patterns (Fig. 1C).

### Numerical model for DMF

To demonstrate the flexibility of the proposed electric field force-based manipulation method, experiments were performed to manipulate hydrogel droplets and cells or microparticles. An electrical signal with an amplitude of 60 V and a frequency of 1 kHz was applied to the individually addressable electrodes, driving a hydrogel droplet to this electrode through the EWOD. Then, the amplitude and frequency of the electrical signal were changed to 15 V and 20 kHz, respectively, before the signal was applied to the electrode. This change in conditions drove the microparticles within the hydrogel droplets to be gathered by the DEP force (Movie S1). The EWOD force driving the hydrogel droplet in a parallel-plate DMF can be described as follows [55]:

$$F_{\text{EWOD}}=\frac{\varepsilon_{0}\varepsilon_{D}L}{2d}{\mathrm{VD}}^{2}$$ (1)

where *ε*_0_ is the permittivity of vacuum, *ε*_D_ is the permittivity of the dielectric layer, *L* is the width of the addressable electrode, *d* is the thickness of the dielectric layer, and *VD* is the voltage of the dielectric layer.

The DEP force acting on a microparticle in hydrogel droplets can be described as follows [56]:

$$F_{\mathrm{DEP}}={\pi \varepsilon }_{0}\varepsilon_{m}r^{3}\text{Re}\left[K\left(\omega \right)\right]\nabla E^{2}$$ (2)

where *ε*_m_ is the permittivity of the hydrogel droplet, *r* is the radius of the cell or microparticle, ∇*E*^2^ is the gradient of the square of electric field strength, and Re[*K*(*ω*)] is the real part of the Clausius–Mossotti (CM) factor, which is described as follows:

$$\text{Re}\left[K\left(\omega \right)\right]=\text{Re}\left[\frac{\varepsilon_{p}^{*}-\varepsilon_{m}^{*}}{\varepsilon_{p}^{*}+2\varepsilon_{m}^{*}}\right]$$ (3)

where *ε*^∗^ = *ε* − *jσ*/*ω*, and *ε* and *σ* are the permittivity and conductivity, respectively. The subscripts *p* and *m* denote the cell or microparticle and the hydrogel droplet, respectively. In addition, *ω* = 2*πf*, where *f* is the frequency of applied voltage.

**Fig. 2.**
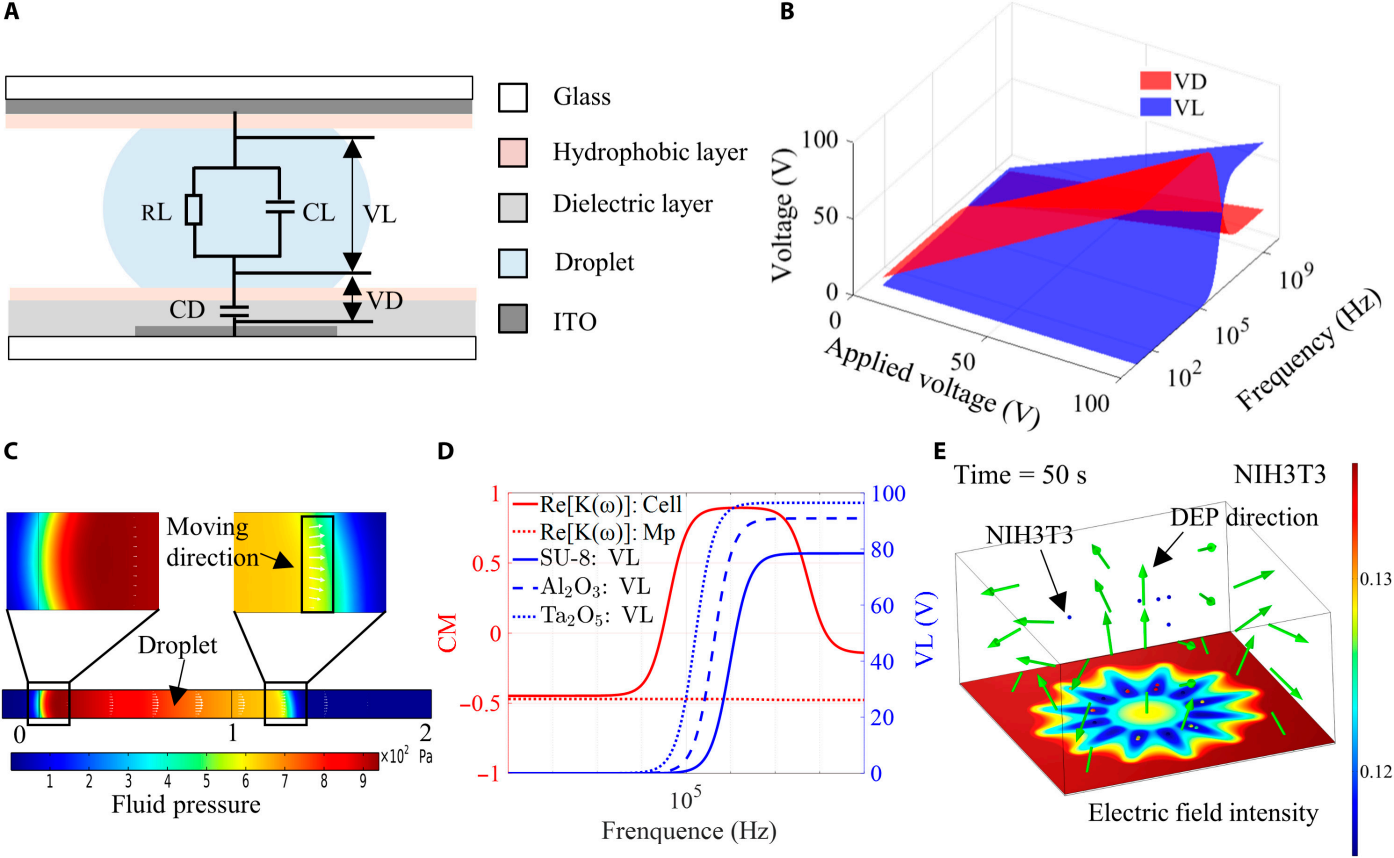
Numerical Analysis of DMF. (A) Cross-sectional view and equivalent circuit model of a parallel-plate DMF device. (B) Voltage distribution in DMF with SU-8 as the dielectric layer. (C) EWOD drives hydrogel droplet movement. (D) Re(*K*(*ω*)) of cells and microspheres and VL of hydrogel droplets. (E) Simulation of DEP in mammalian cells.

To further analyze the performance of EWOD manipulation for hydrogel droplets and DEP manipulation for cells or microparticles, the EWOD and DEP profiles were simulated using a 3-dimensional model in COMSOL Multiphysics (Fig. S1A and B). A corresponding cross-section of a parallel plate of DMF containing hydrogel droplets is shown in Fig. 2A. Based on this cross-section, a simplified equivalent circuit diagram of the DMF was proposed. The figure shows that the applied voltage is primarily distributed across the droplet and dielectric layer, where *VL* is the droplet voltage and *VD* is the dielectric layer voltage. According to Eqs. 1 and 2, the EWOD force is closely related to the voltage *VD*, and the DEP force is closely related to the electric field generated by the voltage *VL*. Therefore, we first simulated the voltage distribution on DMF when 3 different dielectric materials were used as dielectric layers. The resulting distributions of the voltage VD and the voltage *VL* are shown in Fig. 2B (Fig. S2A and B). As the amplitude of the applied voltage increases, the voltages *VD* and *VL* gradually increase. In the low-frequency range, the *VD* exhibits a larger voltage, whereas the *VL* shows a larger voltage in the high-frequency range. As shown in Eq. 1, the larger the *VD* is, the greater the EWOD force. However, considering the saturation of the contact angle and the dielectric strength of the dielectric layer, *VD* cannot be excessively large. Therefore, we selected an applied voltage of 60 V and a frequency of 1,000 Hz to analyze EWOD. Under these conditions, the voltage *VD* on the right electrode is 59.68 V, leading to a reduced contact angle on the right side of the hydrogel droplet. This effect generates a pressure gradient within the hydrogel droplet, driving it to move right, as depicted by the white arrow of Fig. 2C (which indicates the droplet velocity). Based on the simulation analysis, we conclude that EWOD can occur regardless of the dielectric material used, provided that the appropriate voltage amplitude and frequency are chosen to ensure the appropriate voltage *VD*. However, implementing DEP on DMF becomes more challenging due to the presence of a dielectric layer. This challenge arises because of the existence of the dielectric layer, which causes the frequency of the applied voltage not only to affect the CM factor (that determines the positive and negative DEP of the cells or microparticles) but also to affect the voltage *VL* on the hydrogel droplet (which influences the magnitude of the DEP force on the cells or microparticles). Thus, we selected 2 commonly used microparticles and cells, polystyrene microspheres, and mammalian cells and calculated their CM factors in hydrogel droplets. The results showed that the CM factor of polystyrene microspheres in hydrogel droplets remained consistently negative, while the CM factor of mammalian cells transitioned from negative to positive at kilohertz frequencies (Fig. 2D). Next, by combining the simulation results of the voltage *VL* with the CM factor of the microparticle and cell, the results show that when using SU-8 and Al_2_O_3_ as the dielectric layer, the voltage *VL* remains minimal before the CM factor of the mammalian cells becomes positive. However, when Ta_2_O_5_ was used as the dielectric layer, the voltage *VL* amplitude was approximately 2.4 V. Under these conditions, negative DEP was simulated for mammalian cells. The simulation results for negative DEP in mammalian cells are shown in Fig. 2E (Fig. S3A and B). The results indicated that the voltage *VL* was too low to induce negative DEP in mammalian cells, which was in favorable qualitative agreement with the experimental observations. Therefore, when using DEP for cell or microparticle manipulation in DMF, it is necessary to consider a suitable voltage and frequency, as well as the selection of an appropriate dielectric layer material. Based on the above simulation analysis, we can efficiently select the appropriate parameters to achieve positive and negative DEP as well as EWOD manipulation of DMF.

### Construction of hydrogel microstructures on chip

The hydrogel microstructures were constructed within DMF using a systematic process. First, hydrogel droplets were loaded into the chambers of DMF. These droplets were manipulated to move to their designated positions and were then exposed to a UV light pattern. The UV light pattern, projected through a highly transparent ITO-coated glass on the top plate of the DMF chip, triggered the polymerization of the hydrogel droplet, resulting in the immediate formation of the corresponding hydrogel microstructures. As a result, these hydrogel microstructures adhere to the top plate due to the superior adhesion of the hydrophobic coating 3-(trimethoxysilyl) propyl methacrylate (hydrophobic contact angle about 100°) on the top plate compared to the hydrophobic Teflon coating (hydrophobic contact angle about 115°) on the bottom plate [57]. Any residual hydrogel was carefully removed, and the hydrogel microstructures were washed in this position using a wash droplet. Next, a new hydrogel droplet was transferred to the same position using EWOD. Again, the predesigned UV light pattern was projected onto the hydrogel droplet, causing it to polymerize and form new hydrogel microstructures. This sequential process allowed for the creation of heterogeneous hydrogel microstructures with precise control of their geometry, component, and cell type distribution.

**Fig. 3.**
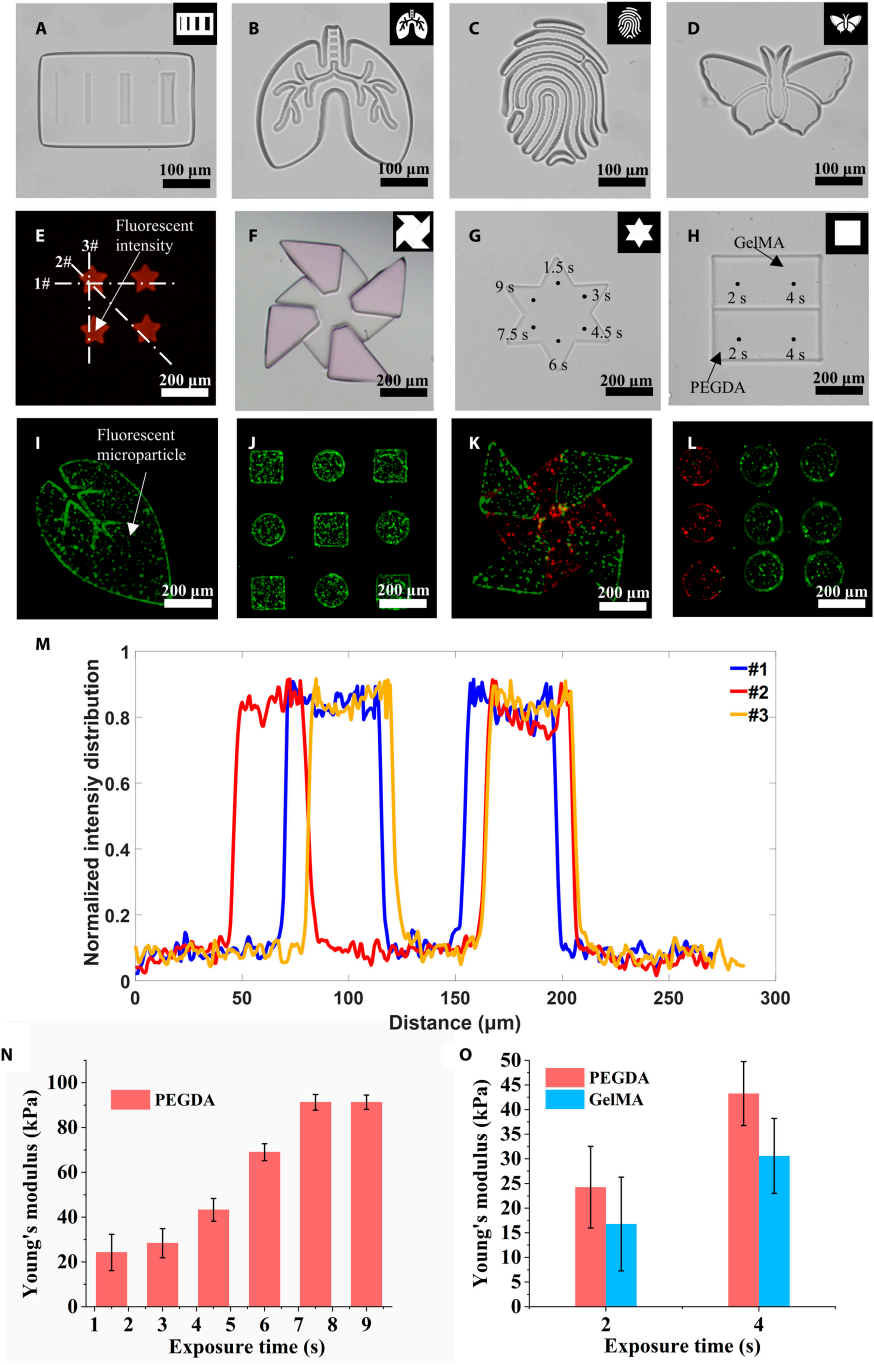
Construction of hydrogel microstructures on a DMF. (A to D) Complex hydrogel microstructures were constructed on a DMF. (E) Matrix of pentagram-shaped hydrogel microstructures incorporating red fluorescent dye. (F) Kite-shaped hydrogel microstructures incorporating composite materials. (G and H) Hydrogel microstructures after different exposure durations. (I to L) Fluorescent microparticles were incorporated into hydrogel to construct hydrogel microstructures that encapsulate fluorescent microparticles. (M) The quantitative fluorescence intensity distribution of 3 curves on the array of pentagram-shaped hydrogel microstructures was visualized using ImageJ. (N and O) Quantitative analysis of Young’s modulus for different exposed regions of the hydrogel microstructures in (G) and (H) was performed using atomic force microscopy.

The resolution of the hydrogel microstructures was evaluated by designing specific patterns. This evaluation process began with the creation of a gradient linear microstructure (Fig. 3A), which exhibited well-defined microgrooves with an approximate width of 10 μm. Furthermore, complex-shaped hydrogel microstructures resembling a lung, fingerprint, or butterfly morphology (Fig. 3B to D) were constructed with remarkable precision, and these microstructures exhibited well-defined shapes and sharp edges. To further examine the homogeneity of the hydrogel microstructures, a pentagram-shaped array of fluorescent hydrogel microstructures was constructed by incorporating a fluorescent dye into the hydrogel droplet (Fig. 3E). Three randomly selected lines within the hydrogel microstructure array were analyzed, and the corresponding fluorescence intensity for each line was plotted (Fig. 3M). These lines exhibited similar fluorescence intensities, demonstrating the uniformity of the hydrogel microstructure array. The fabrication process allowed for precise control of the shape and material distribution of the hydrogel microstructures, as illustrated by the example of a dragon-shaped microstructure (Fig. 3F). This result highlights the versatility and accuracy of the technique in producing complex microstructures. Furthermore, various hydrogel microstructures containing fluorescent microparticles, including single microstructures (Fig. 3I), composite microstructures (Fig. 3K), single array microstructures (Fig. 3J), and composite array microstructures (Fig. 3L), were successfully constructed. The successful construction of these high-precision hydrogel microstructures with complex shapes, a regional distribution of hydrogel material, and microparticle types demonstrated the reliability of the on-chip constructed microstructures.

Previous studies have demonstrated that the UV exposure dose impacts the distribution of Young’s modulus on hydrogel microstructures [58,59]. In this study, to optimize the Young’s modulus of the hydrogel microstructures, the exposure time for various exposure areas was dynamically adjusted without changing the light intensity, thereby enabling the adjustment of different UV exposure doses in those areas. For example, the hexagonal star-shaped hydrogel microstructure was fabricated with varying exposure times (1.5, 3, 4.5, 6, 7.5, and 9 s) in each corner region, as shown in Fig. 3G (Movie S2). In addition, square composite hydrogel microstructures composed of PEGDA and GelMA hydrogels were exposed for selective exposure times of 2 and 4 s, as shown in Fig. 3H. After biofabrication, the Young’s modulus of both the hexagonal star hydrogel microstructure and the square composite hydrogel microstructure were measured using atomic force microscopy. The Young’s modulus for each area was calculated by averaging the 5 measurements (Fig. 3N and O). The distribution of Young’s modulus in different regions of the hexagonal star hydrogel microstructure is shown in Fig. 3N. As the exposure time increased to 6 s, the Young’s modulus exhibited a progressive increase. However, after 6 s, the Young’s modulus of the hydrogel microstructure remained stable, indicating the completion of the cross-linking reaction and the cessation of any further changes caused by excess light exposure. The Young’s modulus test values for the square composite microstructure comprising the PEGDA hydrogel and the GelMA hydrogel are shown in Fig. 3O. Notably, the Young’s modulus of the PEGDA hydrogel microstructure and the GelMA hydrogel microstructure showed significant differences at identical exposure times, and this difference increased with increasing exposure times. These experiments highlighted that the system is capable of not only constructing various high-precision hydrogel microstructures with complex shapes, regional distributions of hydrogel materials and microparticle types but also regulating the distribution of Young’s modulus within these microstructures during the fabrication process.

### Hydrogel microstructures with a gradient distribution of microparticles

**Fig. 4.**
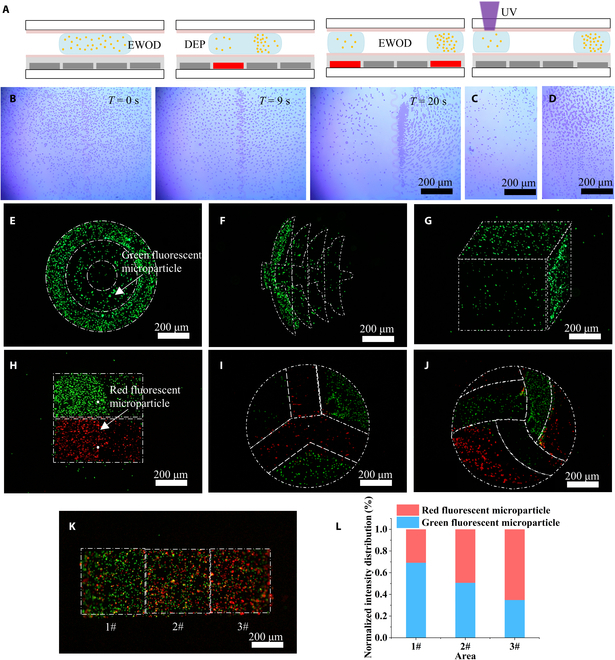
Adjustment of microparticle density within hydrogel droplets on DMF and construction of hydrogel microstructures with microparticle gradient distributions. (A) Schematic of the process of manipulating hydrogel droplets, microparticles with EWOD and DEP, and UV curing of hydrogel droplets. (B) Adjustment of microparticle density in hydrogel droplets with DEP. (C and D) Microparticle density distribution in 2 daughter droplets (E to G) Construction of hydrogel microstructures with different microparticle density gradients. (H to J) Construction of hydrogel microstructures with composite microparticle density gradients. (K) Construction of hydrogel microstructures with mixed microparticle density gradients. (L) Quantitative analysis of the distribution of the different fluorescent microparticles within the hydrogel microstructures.

Under the controlled application of electrical signals, microparticles within a hydrogel droplet are manipulated using DEP. The migration of microparticles (from the left to the right side of the hydrogel droplet) is driven by DEP, as shown in Fig. 4A. Subsequently, the hydrogel droplet is split into 2 subdroplets using EWOD, allowing adjustment of the microparticle density within the hydrogel droplet (Movie S3). Similarly, the merging of 2 subdroplets also alters the microparticle density within the hydrogel droplet. The hydrogel droplet with controlled microparticle density is then cured by UV, resulting in the formation of hydrogel microstructures encapsulating microparticles with different densities. The process for adjusting the microparticle density of the polystyrene microspheres is shown in Fig. 4B. The initial density distribution started at *T* = 0 s, followed by gradual aggregation from left to right, which was driven by negative DEP. Finally, the density distribution was determined after the hydrogel droplet split into 2 subdroplets, as shown in Fig. 4C and D. The microparticle density of one of the subdroplets decreased in comparison to the density of the mother microdroplet, as shown in Fig. 4C. Conversely, the microparticle density of the other subdroplets increases in comparison to the density of the mother droplet, as shown in Fig. 4D. Therefore, the integration of EWOD and DEP on DMF enables efficient and noncontact adjustment of the microparticle density within hydrogel droplets.

Furthermore, shape-controllable hydrogel microstructures with various microparticle densities (Fig. 4E to K) were constructed using DLP to photopolymerize hydrogel droplets with adjusted microparticle density. In Fig. 4E to G, the gradient distribution of single fluorescent microparticles within the hydrogel microstructures is demonstrated. Specifically, as shown in Fig. 4E, the density of fluorescent microparticles within the circular hydrogel microstructure gradually increased in the radial direction (Movie S4). As shown in Fig. 4F, the hydrogel microstructure, shaped like the logo graphic of the Beijing Institute of Technology, exhibited a gradual decrease in the density of fluorescent microparticles from left to right. Finally, as shown in Fig. 4G, a planar cubic hydrogel microstructure with varying densities of fluorescent microparticles on each of its 3 surfaces was demonstrated. In Fig. 4H to J, the density gradient distribution of green and red fluorescent microparticles within the composite hydrogel microstructure was shown. Specifically, as shown in Fig. 4H, in the square composite hydrogel microstructure, the green fluorescent microparticles were located at the upper side, with their density decreasing from left to right, while the red fluorescent microparticles were located at the lower side, exhibiting a similar density trend as the green fluorescent microparticles. As shown in Fig. 4I, within the circular composite hydrogel microstructure, the green fluorescent microparticles exhibited a gradient density distribution within 3 fan-shaped regions, whereas the red fluorescent microparticles displayed a gradient density distribution within rectangular and V-shaped regions. As shown in Fig. 4J, in the circular composite hydrogel microstructure, the green fluorescent microparticles demonstrated a gradient density distribution in the upper 3 regions, while the red fluorescent microparticles demonstrated a gradient density distribution in the lower 3 regions. A hydrogel microstructure with a density gradient of mixed microparticles is shown in Fig. 4K. In this microstructure, the density of the red fluorescent microparticles increased from left to right, while that of the green fluorescent microparticles decreased from left to right. The mixed microparticle density was further quantified by normalizing the fluorescence intensity, as shown in Fig. 4L. The density of microparticles in hydrogel droplets can be adjusted through the combined operation of EWOD and DEP. Hydrogel microstructures were constructed through DLP to photopolymerize hydrogel droplets. This method enables the construction of composite hydrogel microstructures with complex shapes, a regional distribution of hydrogel material, and microparticle type/density.

### Hydrogel microstructures with microparticle patterns

**Fig. 5.**
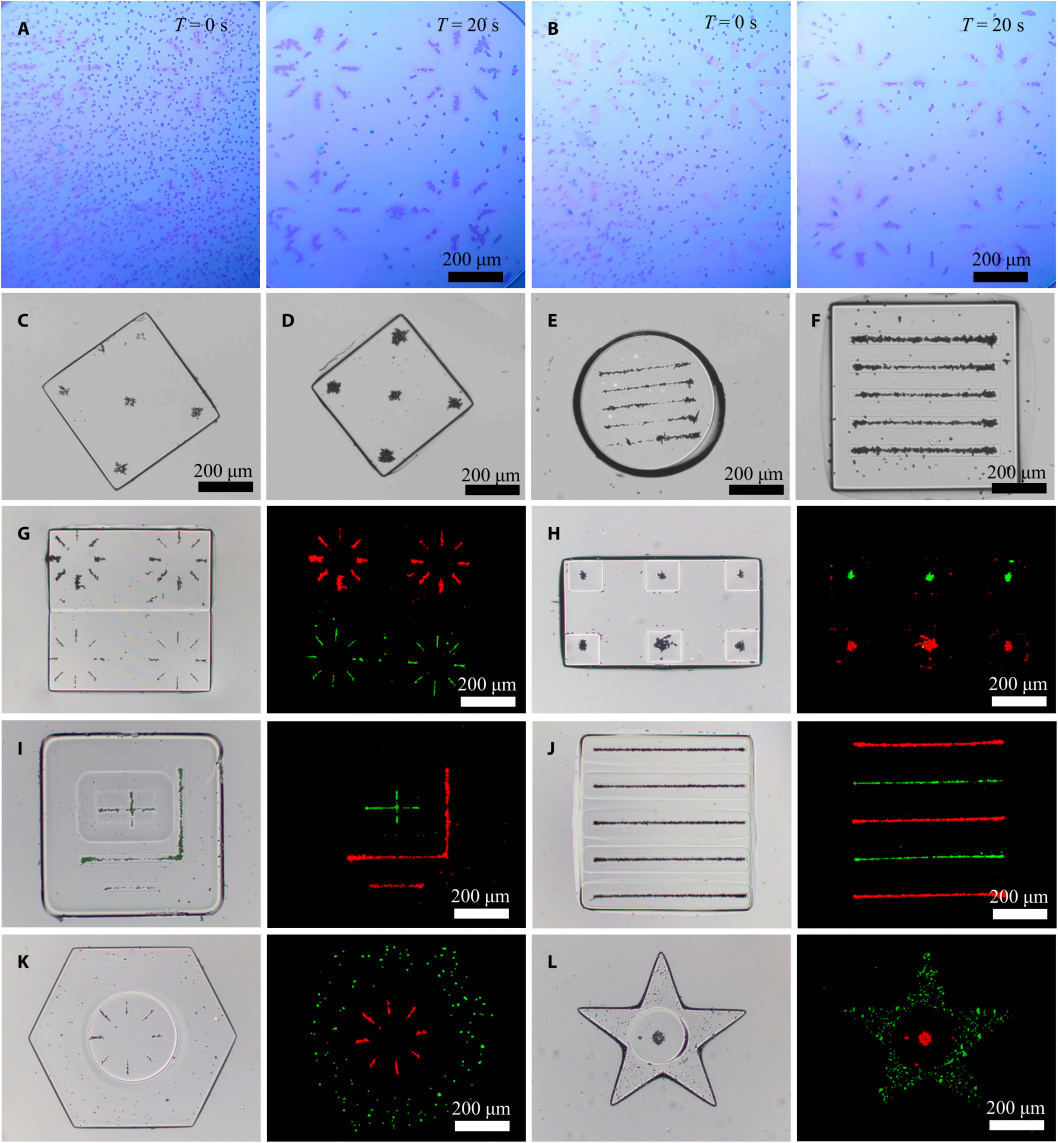
The construction of hydrogel microstructures with internally encapsulated microparticle patterns was achieved on DMF. (A and B) Microparticle patterns were formed within hydrogel droplets of varying microparticle density. (C to F) Hydrogel microstructures encapsulating microparticle patterns. (G to J) Patterns of different types of microparticles were encapsulated in hydrogel microstructures. (K and L) Microparticles and microparticle pattern were simultaneously encapsulated in the hydrogel microstructure.

The integrated system enables the regulation of microparticle density within hydrogel droplets to construct a hydrogel microstructure with controllable microparticle density. However, the microparticles within the hydrogel microstructure are gradient distributed and cannot form microparticle patterns. To address this limitation, an addressable electrode was further patterned through wet etching. This operation allowed the selective formation of microparticle patterns in specific regions using DEP. As described in the previous section, 2 hydrogel droplets with different microparticle densities were generated through the cooperative operation of EWOD and DEP. As shown in Fig. 5A and B, microparticles within the 2 hydrogel droplets gradually aggregated to form microparticle patterns under the influence of DEP (Movie S5). Notably, there was a significant difference in the size of the microparticle patterns between the hydrogel droplets. This further demonstrated that the integrated system is capable of not only regulating the microparticle density within hydrogel droplets but also forming localized microparticle patterns of different sizes.

Furthermore, hydrogel droplets containing microparticle patterns underwent photopolymerization via DLP to construct shape-controllable hydrogel microstructures encapsulating microparticle patterns (Fig. 5C to L). As shown in Fig. 5C and D, 2 square hydrogel microstructures contained microparticle patterns of different sizes while maintaining the same shape. Similarly, as shown in Fig. 5E and F, both the square and round hydrogel microstructures contained microparticle patterns of identical sizes and shapes. As shown in Fig. 5G and H, the composite hydrogel microstructures contained various microparticle patterns of the same size and shape. As shown in Fig. 5I and J, the composite hydrogel microstructure contains linear patterns of different kinds of microparticles that cross each other to form a complex pattern. Finally, hydrogel microstructures with composite attributes, including mixed shape, microparticle type, density, and pattern, was constructed (Figs. 5K and L). As shown in Fig. 5K, a hexagonal hydrogel microstructure nests with a circular hydrogel microstructure. The hexagonal microstructure contained uniformly distributed green fluorescent microparticles, while the circular hydrogel microstructure contained radial red fluorescent microparticles. As shown in Fig. 5L, a pentagram-shaped hydrogel microstructure nests with a circular hydrogel microstructure. The pentagram hydrogel microstructure contained uniformly distributed green fluorescent microparticles, while the circular hydrogel microstructure contained spherical red fluorescent microparticles. These results demonstrated the versatility of the integrated system, enabling the construction of microstructures with different shapes, microparticle densities, and various microparticle patterns.

### Biological assessment of cell-laden hydrogel microstructures

**Fig. 6.**
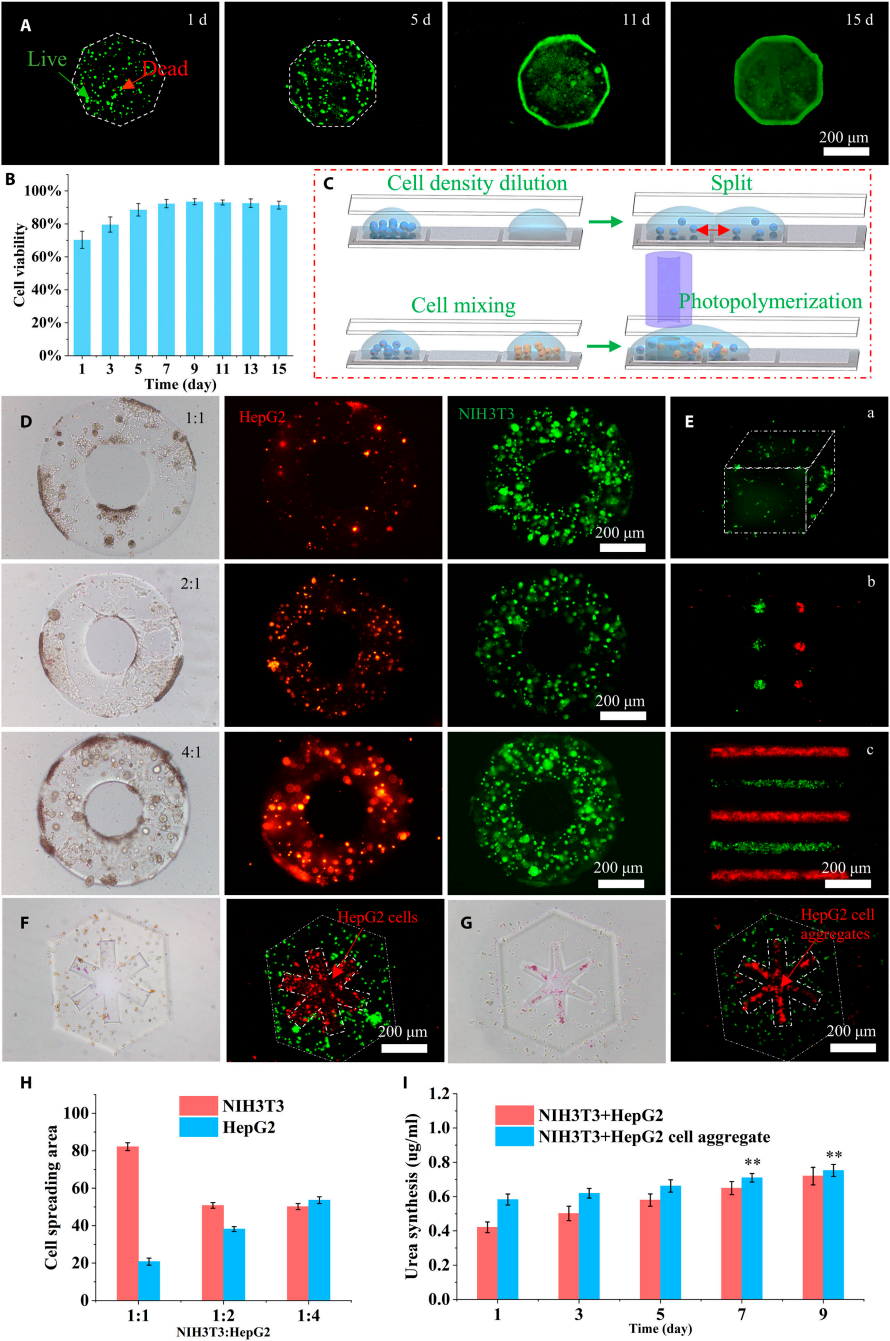
The hydrogel microstructures were constructed via the internal encapsulation of cells in DMF, after which the biological functionality of the hydrogel microstructures was evaluated. (A) Cells encapsulated in hydrogel microstructures were stained with calcein acetoxymethyl ester (green, live cells) and propidium iodide (red, dead cells) after culturing for 1, 5, 11, and 15 d. (B) Demonstration of the viability of cells encapsulated in hydrogel microstructures. (C) The process diagram comprises 3 key steps: cell density regulation, mixing of various cell types, and construction of hydrogel microstructures encapsulating cells. (D) Different density ratios of HepG2 cells and NIH3T3 cells were encapsulated in hydrogel microstructures and cocultured for 1 week to observe the proliferation of both cells (HepG2 cells were stained red, and NIH3T3 cells were stained green by PKH26 and PKH67 [Sigma-Aldrich]). (E) Fluorescence images of density gradients of NIH3T3 cells encapsulated in hydrogel microstructures, HepG2 cell aggregates, and NIH3T3 cell aggregates patterned for encapsulation in hydrogel microstructures. (F) Bright-field and fluorescence images of HepG2 cells and NIH3T3 cells encapsulated in hydrogel microstructures to construct a hepatic lobule model. (G) Bright-field and fluorescence images of HepG2 cell aggregates and NIH3T3 cells encapsulated in hydrogel microstructures to construct a hepatic lobule model. (H) Demonstration of the area of proliferation of HepG2 cells and NIH3T3 cells in coculture. (I) Demonstration of urea secretion by HepG2 cells and NIH3T3 cells cocultured in hydrogels encapsulated in different forms. The values represent the means of 3 independent experiments ^^∗^∗^*P* < 0.01.

In vitro, the construction of hydrogel microstructures with precise control of their shape, ECM, cell density, and cell pattern to replicate real tissue microstructures with high biomimicry offers immense potential for applications in the field of functional tissue engineering. For example, such composite hydrogel microstructures may be suitable for broad applications in drug modeling, drug delivery, and tissue repair. To examine cell growth within the hydrogel microstructure, we incubated the octagonal hydrogel microstructures encapsulating NIH3T3 cells in a biological incubator. Cell viability was assessed by using live/dead staining after 15 d of culture (Fig. 6A). As shown in Fig. 6B, cell viability was approximately 70% on the first day of culture. Over time, the cell viability steadily increased, indicating that the hydrogel microstructures constructed through the system did not adversely affect cell activity. As shown in Fig. 6C, the HepG2 cell density within the hydrogel droplet was adjusted. Subsequently, the hydrogel droplet was mixed with another hydrogel droplet containing NIH3T3 cells and then photopolymerized to construct hydrogel microstructures that incorporated both cell types. Different initial densities of HepG2 cells were incorporated into these microstructures. For these hydrogel microstructures, NIH3T3 cells were labeled with green fluorescence, and HepG2 cells were labeled with red fluorescence. Bright-field and fluorescence images taken on day 7 of hydrogel microstructure culture are shown in Fig. 6D. Furthermore, the quantitative analysis of the proliferation rate of the 2 cell types within the hydrogel microstructure is shown in Fig. 6H. Notably, HepG2 cells exhibited a slower proliferation rate than NIH3T3 cells. Therefore, when the cells are cultured together, regulating their initial density is crucial for achieving the desired cell distribution. We further constructed hydrogel microstructures with a gradient of NIH3T3 cell densities and patterned distribution of NIH3T3 and HepG2 cell aggregates, as shown in Fig. 6E (a to c). These hydrogel microstructures underscore the functionality of our biofabrication technology. Additionally, 2 different biomimicry microstructure models of the hepatic lobule were constructed, as shown in Fig. 6F and G. The first model involved coculturing NIH3T3 cells and HepG2 cells within hydrogel microstructures. In contrast, the second model included NIH3T3 cells and the ordered arrangement of HepG2 cells within hydrogel microstructures. Urea secretion was measured in these biomimicry microstructure models of the hepatic lobule during 2 cultivation cycles. As shown in Fig. 6I, the second model exhibited significantly greater urea secretion than the first model. This difference indicates that the order of arrangement of cell states within hydrogel microstructures affects the functional expression of microstructure models. Thus, these microstructures provide a platform for constructing biomimetic biological microstructures that enable study of cell behavior and interactions within specific microenvironments, facilitating a deeper understanding of cell signaling mechanisms. The potential applications of these microstructures are proposed to drive further development and innovation in related fields, particularly in medicine and related disciplines.

## Discussion

In this study, we developed a novel approach for constructing tissue-mimicking microstructures. The proposed strategy combines cross-scale manipulation of macroscopic hydrogel droplets and microscopic cells with high-precision modulable encapsulation to yield various hydrogel microstructures. First, various cell-laden hydrogel droplets are driven to designated electrodes using EWOD. Second, the cells in the hydrogel droplets are driven to specific locations using DEP. Third, a UV beam modulated with a DMD is used to form an intended shape, which is projected onto the hydrogel droplets. This excitation triggers selective photopolymerization of the hydrogel droplets, resulting in the construction of the desired hydrogel microstructures. The generated hydrogel microstructures replicate the ECM geometry and component, cell type, and density and pattern of natural tissues and are therefore potentially applicable as novel pathological or pharmacological models. Moreover, this strategy is proposed to realize more complex microstructures than those demonstrated here, such as hepatic lobules, islets, and kidneys, with physiological significance.

## Materials and Methods

### Experimental setup

The DMF device consists of core components, including a DMF chip, a microcontroller unit, a signal generator (33600A, KEYSIGHT), a voltage amplifier (ATA-2021B, Aigtek), and a high-voltage ac switching matrix. The signal generator generates an ac signal, which is then amplified by the voltage amplifier before being sent to the high-voltage ac switching matrix. The switching matrix is connected to the electrodes of the DMF. Coordinated with instructions from the host computer, the microcontroller generates precise digital signals that control the switching mechanism within the high-voltage ac switching matrix, enabling seamless activation and deactivation of the electrodes on the DMF. By applying appropriate electrical signals to these electrodes, we performed targeted EWOD and DEP operations to achieve controlled manipulation of hydrogel droplets and microparticles.

Dynamic optical projection lithography consists of several key components, including a UV light source (5 W, 405 nm; Shanghai Runcast Electronic Technology Co., Shanghai, China), beam expander optics, DMD (V-7000VIS, TX, USA), projection optics and a charge-coupled device. The emitted UV light beam is directed through the beam expander optics to adjust its diameter. This modified light beam is then projected to the DMD, which is responsible for reflecting a patterned light beam aligned with the input design mask. The patterned light beam, now refined, travels through the projection optics onto a hydrogel droplet, resulting in its controlled photopolymerization process.

### Hydrogel ink material

A PEGDA hydrogel was prepared by dissolving PEGDA (Mn 3400; Laysan Bio, USA), acrylate-PEG-RGDS (JenKem Tech, USA) and pluronic F127 (Sigma-Aldrich, St. Louis, MO USA) in low-conductive buffer (LCB) (10 mM Hepes, 0.1 mM CaCl_2_, 59 mM D-glucose, and 236 mM source, pH 7.35). A 50% (w/v) solution of photoinitiator was prepared by dissolving 2-hydroxy-1-(4-(hydroxyethoxy)phenyl)-2-methyl-1-propanone (Irgacure 2959, BASFSE, Germany) in dimethyl sulfoxide (Fisher Scientific, USA) and adding it to the PEGDA hydrogel to be used as a photoinitiator. The final concentrations of the components in the PEGDA hydrogel were 15% w/v for PEGAD, 0.5% w/v for photoinitiator, 0.1 w/v for pluronic F127, and 5 mM RGDS in the LCB.

A GelMA hydrogel was prepared by dissolving GelMA prepolymer (Suzhou Yongqinquan Intelligent Equipment Co., Suzhou, China), pluronic F127 (Sigma-Aldrich), and lithium phenyl-2,4,6-trimethyl-benzoylphosphinate (LAP, Allevi, USA) into the LCB. The final concentrations of the components in the GelMA hydrogel were 5% w/v for GelMA, 0.5% w/v for LAP, and 0.1% for pluronic F127 in the LCB.

### Cell cultures

HepG2 (human hepatocellular carcinoma) and NIH3T3 (mouse fibroblast) cells were purchased from the American Type Culture Collection (Manassas, VA, USA). HepG2 and NIH3T3 cells were cultured separately in Dulbecco’s modified Eagle’s medium (HyClone, Logan, UT, USA) supplemented with 10% (v/v) fetal bovine serum (Gibco, Gaithersburg, MD, USA) and 1% (v/v) penicillin–streptomycin solution (Solarbio, Beijing, China). The samples were stored in an incubator supplemented with 5% CO_2_ at 37 °C, and the medium was changed every 2 to 3 d. Before encapsulation, HepG2 and NIH3T3 cells were collected separately and resuspended in LCB. To distinguish the distribution of different cell types in the hydrogel microstructure, HepG2 cells were stained red, and NIH3T3 cells were stained green by PKH26 and PKH67 (Sigma-Aldrich).

### Live/dead cell staining

Live/dead staining assays of the cell encapsulation within the hydrogel microstructures were performed by labeling with calcein acetoxymethyl ester (2 μg/ml) and propidium iodide (3 μg/ml) fluorescent dyes (Molecular Probes, Eugene, OR, USA). Cellular viability within the hydrogel microstructures was assessed daily using ImageJ software to analyze the areas of red fluorescence (dead cells) and green fluorescence (live cells) in each image. Cellular viability was quantified by calculating the ratio of the area of green fluorescence relative to the total area of fluorescence.

### Urea assay

For urea secretion, the amount of urea secreted into the medium was measured using a urea assay kit (Sigma-Aldrich). Before the assay, the constructed microstructures were washed with Dulbecco’s modified Eagle’s medium and placed in fresh medium. After 24 h, the medium was collected and temporarily stored at −20 °C. After all the control groups were sampled, the solutions were gradually thawed to room temperature for the assays. The urea concentration was analyzed using a urea assay kit (Sigma-Aldrich). The absorbance was read using a microplate reader.

## Data Availability

The authors confirm that the data supporting the findings of this study are available within the article and/or its supplementary materials.
