# Supplementary material for: Integrated Cross-Scale Manipulation and Modulable Encapsulation of Cell-Laden Hydrogel for Constructing Tissue-Mimicking Microstructures
Source: Research (Wash D C). 2024 Jul 16;7:0414. doi: 10.34133/research.0414 (PMC11266663; doi:10.34133/research.0414)
Supplement: Supplementary 1 — Figs. S1 to S3 Movies S1 to S6 [file research.0414.f1.zip › Supporting Information.docx]

Supporting Information

3D simulations of EWOD were generated in COMSOL Multiphysics using the Fluid Flow module. As shown in Fig. S1 A, the EWOD model length (X-axis), width (Y-axis), and height (Z-axis) are set to 2000 μm, 1000 μm, and 100 μm, respectively. The microdroplet has a radius of 480 μm and a height of 100 μm. The microdroplet has a conductivity of 0.020 S/m and a permittivity of 70.

3D simulations of DEP were generated in COMSOL Multiphysics using the AC/DC module. As shown in Fig. S1 B, the DEP model (X-axis), width (Y-axis), and height (Z-axis) are set to 200 μm, 200 μm, and 100 μm, respectively. The bottom of the model is a 2 μm dielectric layer material. The microparticles are one of the mammalian cells and polystyrene microspheres. The electrical parameters for mammalian cells are as follows: cell membrane conductivity is 3E-6 S/m and a permittivity is 8, the cytoplasm has a conductivity of 0.5 S/m and a permittivity of 50, the radius is 4.0 μm, the membrane thickness is 8 nm. The electrical parameters of polystyrene microspheres are a dielectric constant of 2.5 and a conductivity of 8E-4 S/m.


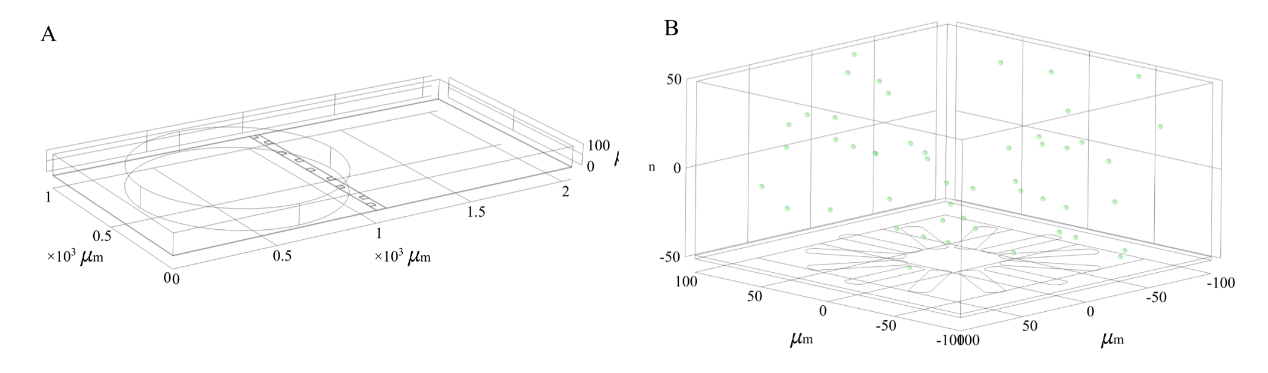


Fig. S1. The simulation model of the EWOD and DEP. (A) The sketch of the 3D simulation model for EWOD. (B) The sketch of the 3D simulation model for the DEP.


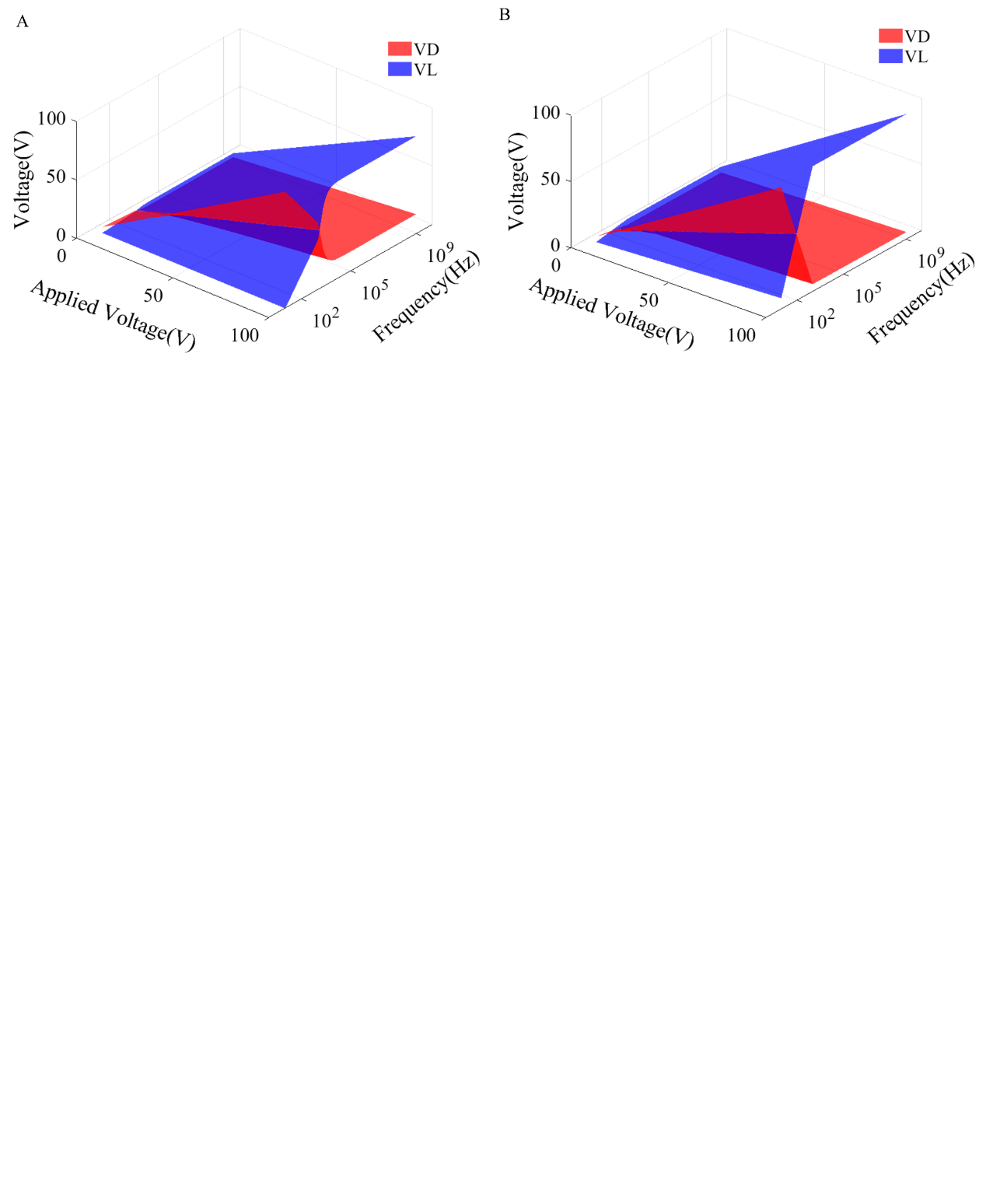


Fig. S2. Voltage distribution in DMF. (A) Voltage distribution in DMF with AL_2_O_3_ as dielectric layer. (B) Voltage distribution in DMF with Ta_2_O_5_ as dielectric layer.


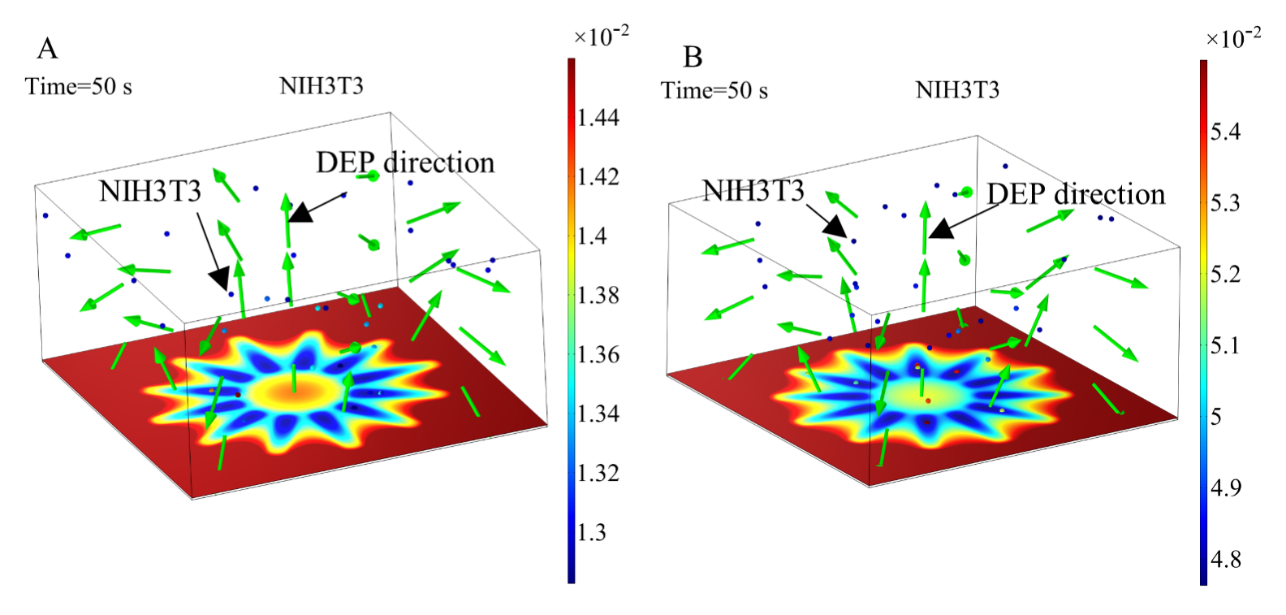


Fig. S3. Simulation of DEP in mammalian cells. (A) The simulation of negative DEP on mammalian cells with SU-8 as dielectric layer. (B) The simulation of negative DEP on mammalian cell with Al_2_O_3_ as dielectric layer.
